# Mitochondrial DNA analysis reveals spatial genetic structure and high genetic diversity of *Massicus raddei* (Blessig) (Coleoptera: Cerambycidae) in China

**DOI:** 10.1002/ece3.6799

**Published:** 2020-10-01

**Authors:** Yufan Zhang, Atif Manzoor, Xiaoyi Wang

**Affiliations:** ^1^ Key Laboratory of Forest Protection of National Forestry and Grassland Administration Research Institute of Forest Ecology, Environment and Protection Chinese Academy of Forestry Beijing China

**Keywords:** genetic differentiation, genetic structure, geographical population, *Massicus raddei*, mitochondrial DNA, outbreak

## Abstract

The oak longhorned beetle (OLB), *Massicus raddei* (Blessig, 1872) (Coleoptera: Cerambycidae), is widely distributed in Asia (China, the Korean Peninsula, Japan, Vietnam and the Russian Far‐East), but pest outbreaks have occurred only in Liaoning Province and Jilin Province of China. In order to explore possible mechanisms of local population outbreaks and characterize the genetic diversity and genetic structure of *M. raddei* across its range in China, three mitochondrial genes (*COI, Cytb,* and *COII*) were sequenced and analyzed for seven *M. raddei* populations collected from six provinces in China. From these different populations, we found a high haplotype and nucleotide diversity. Haplotype networks and phylogenetic analyses both demonstrate apparent genetic diversification between SC (southern China) and NC (northern China) population groups. A set of 21 pairwise comparisons for *Fst* (pairwise fixation indices) and *Nm* (genetic flow index) showed significant genetic differentiation and limited gene flow except for two pairs, Shandong (SD) and Liaoning (LN), and Anhui (AH) and Henan (HN). This pattern suggested that the periodic outbreak of the LN population could not be attributed to the absence of genetic flow with other spatial populations and that regional environmental factors might be responsible. AMOVA (Analysis of molecular variance) showed that the greater molecular genetic variation was among populations. Based on Tajima's *D* statistic, Fu's *Fs*, and the mismatch distribution test, we determined that the seven populations sampled were stable and had not experienced any recent population expansion. The fact that all the sampled populations showed only unique haplotypes and lacked shared or ancestral haplotypes, as well as the nonstar‐like distribution of haplotype network for concatenated genes, collectively provided powerful evidence of the stable and isolated nature of most populations. The high genetic differentiation and spatial genetic structuring among populations are both likely related to the beetle's moderate flight capacity, regional variation in host tree species and microclimate, as well as the geographic distance between sampling sites.

## Graphical abstract

The present study used three genes of mitochondrial DNA to explore the patterns of genetic structure among seven populations of *Massicus raddei* across the natural distribution of this species in China. High genetic diversity and distinct spatial genetic structuring were identified through the mitochondrial analyses, and the assumption that particular periodic behaviors and outbreaks of *M. raddei* in northeastern China mainly result from the absence of gene flow and local isolation was preliminarily denied.

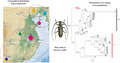

## 1 INTRODUCTION

*Massicus raddei* (Blessig) (Coleoptera: Cerambycidae) is known as a serious trunk borer that mainly infests species of oaks (Chen et al., 1959). The larvae of *M. raddei* bore into the xylem of host trees, creating large galleries that reduce the transportation of water and nutrition, causing crown dieback and ultimate death (Sun, 2001). *M. raddei* has a wide distribution in the oriental region, including most provinces in China (Sun, 2001), the Korean Peninsula (Cho et al., 2010; Kim et al., 2014; Lim et al., 2014), Japan (Kojima, 1931; Leksono et al., 2006), Vietnam (Nga et al., 2014), and the Russian Far‐East (Anisimov & Bezborodov, 2017; Mokuroku Database, 2017). Although this borer species has not yet been reported from other continents, there still exists a risk of invasion vectored by transplanted host plants or timbers infested with its larvae or pupae, this species has recently been added to the “alert list” of the European and Mediterranean Plant Protection Organization and the United States Department of Agriculture (EPPO, 2015; USDA, 2015). This borer shows ecological differences in its life history, host trees, and timing of emergence among populations in various regions (EPPO, 2018). In heavily infested stands of *Quercus mongolica* Fisch.ex Leddb and *Quercus liaotungensis* Mary in northeastern China (Liaoning Province), one generation is completed in 3 years with six larval instars (Wang et al., 2012). *M. raddei* was first reported as damaging oak forests in Liaoning Province, with nearly perfectly synchronized mass emergences noted in every 3 years from 1993 to 2017. However, in interval 2 years, there were relatively scarce adults to emerge (Sun et al., 2010; Wang & Shao, 2013; Wang et al., 2012; Yang et al., 2011). In Shandong Province, Anhui Province, and Henan Province, *M. raddei* attacks *Quercus acutissima* Carruth, while in more southern places such as Fujian and Yunnan provinces, the common host is *Quercus glauca* Thunb. In addition, fewer than 3 years might be needed in these sites (EPPO, 2018). In other distributional areas apart from Liaoning and Jilin provinces, *M. raddei* does not display periodic emergence, and adults emerge every year without outbreaks and do not pose great threat to the growth of oak forests (Luo et al., 2005; Wei et al., 2009; State Forestry Administration, 2016; EPPO, 2018). Therefore, there are reasons to suspect that different ecological patterns of phenology and density of *M. raddei* might be influenced by limited genetic flow among spatial populations, variation in host tree adaptation, and regional climatic conditions, as well as the regulatory effect of natural enemies (such as *Dastarcus helophoroide*s Fairmaire or *Cerchysiella raddeii* Yang found in oak forests of Liaoning Province) (Gao et al., 2003; Yang et al., 2013).

The periodicity, involving synchronized adult emergences, fixed life‐cycle length, and intervals between emergences with no or scarce adults present, is rare in insects (Heliövaara et al., 1994). Of these, periodical cicadas *Magicicada* spp. and some periodical moths are typical study objects for deep comprehension of periodicity development (Ito et al., 2015; Martin & Simon, 1990; Várkonyi et al., 2002; Williams & Simon, 1995). Previously, Yoshimura (1997) proposed that limited gene flow restricted by geographical barriers and extreme low temperature events during the Pleistocene might be driving factors for the development of periodical cicadas from nonperiodical species *Okanagana* spp. In addition, the adults of the noctuid genus *Xestia* exhibit periodic dynamics in eastern Lapland but not in western Lapland, Kankare et al. (2002) discovered that substantial genetic differentiation and isolation did not exist between nonperiodical and periodic cohorts, which might not be a plausible explanation for its periodicity evolution. As for *M. raddei*, whether there exist in subtle genetic differentiation and limited gene flow between population of Liaoning Province and those of other distributional regions is still a question, which is speculated to reinforce its development of their particular periodic adult emergence pattern in Liaoning Province.

Recently, mitochondrial DNA markers *COI* (cytochrome oxidase subunit I), *Cytb* (cytochrome b), and *COII* (cytochrome oxidase subunit II) genes have been developed into the common molecular tool for many phylogenetic studies of beetles. Eight microsatellite loci and a partial mtDNA *COI* gene were used as molecular markers to elucidate patterns of genetic structure among 32 populations of the endangered beetle *Rosalia alpina* Linnaeus (Coleoptera: Cerambycidae) across central and south‐east Europe, to clarify its biology and recent population expansion, and to devise more effective conservation measures (Drag et al., 2015). Tang et al. (2016) used the combination of three mitochondrial genes to study the genetic structure of *Galerucella birmanica* Jacoby (Coleoptera: Chrysomelidae) in its main distributional habitats. Moreover, Javal et al. (2017) investigated invasion history of *Anoplophora glabripennis* Motschulsky (Coleoptera: Cerambycidae) across Europe based on *COI* gene. Lee et al. (2020) presented the entire genetic structure among ALB (*A. glabripennis*) populations of South Korea and confirmed that ALB invasion has occurred even within the species’ native ranges based on *COI* mitochondrial sequences. Studies on *M. raddei* have focused mostly on its biology, damage pattern, field surveys for natural enemies, and integrated control measures (Li et al., 2017; Tang, 2011; Wang et al., 2010; Yang et al., 2013, 2014). However, as yet there has been no further researches into genetic aspects of *M. raddei* populations in China. In this paper, we used three mitochondrial *COI, Cytb,* and *COII* genes to investigate the genetic diversity and differentiation among seven geographically separated populations of *M. raddei*, and infer the genetic structure of the sampled populations. We then evaluated whether synchronic pattern of adult emergence of *M. raddei* in Liaoning Province resulted from the lack of genetic flow with other spatial populations and local isolation.

## 2 MATERIALS AND METHODS

### 2.1 Specimen collection

**FIGURE 1:**
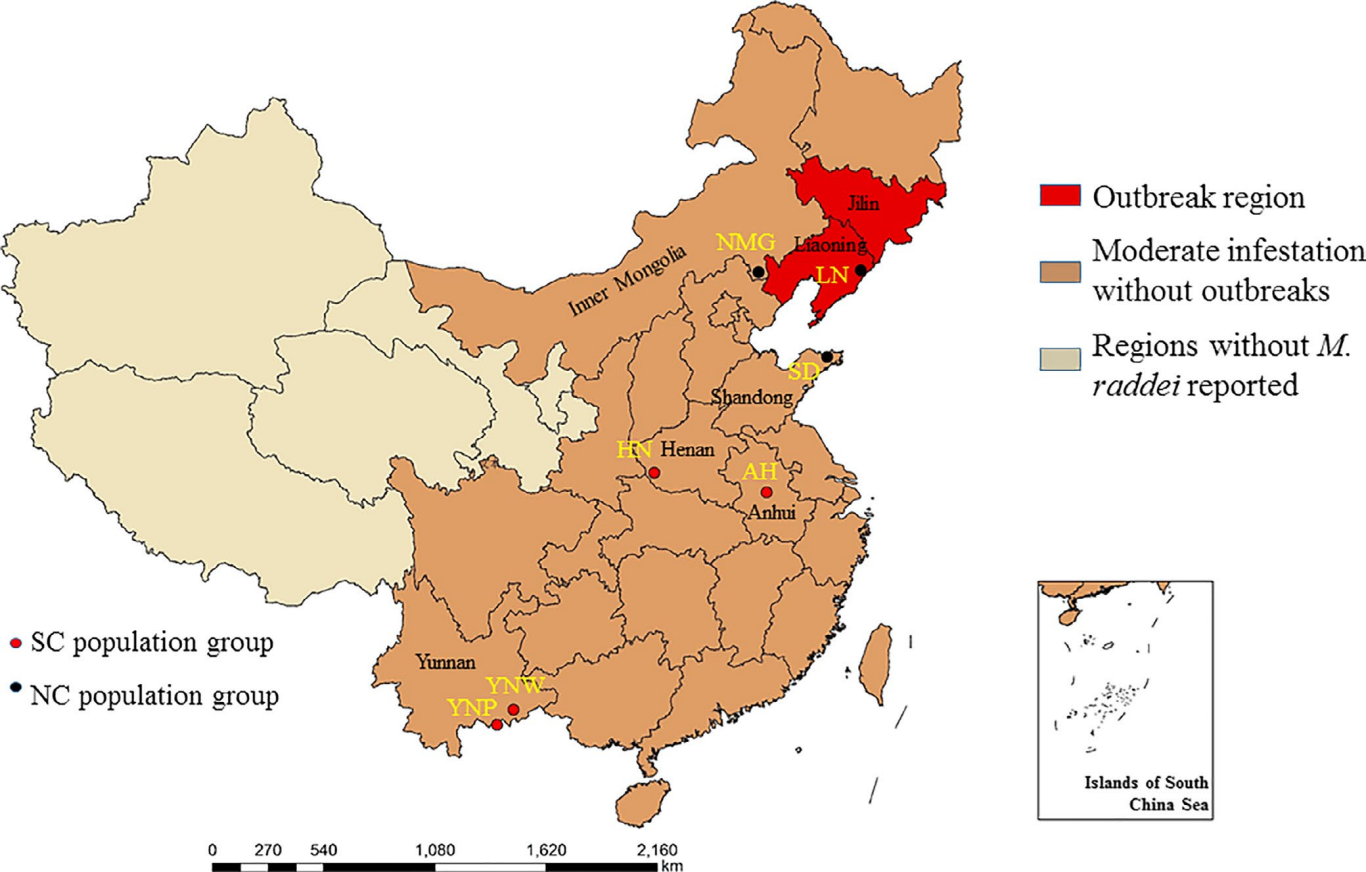
Map illustrating the distribution and sampled localities of *Massicus raddei* (Blessig) in China. NC and SC population groups are labeled by black and red points, respectively. The sampling provinces and outbreak regions are labeled, and the population codes are shown in yellow words

A total of 40 adults of *M. raddei* were collected by a specially designed black light (Jiang et al., 2010) at night from early May to late August in 2019. The seven locations mostly cover the range of this pest which differs in host species in China (Figure 1; Table 1). The observation of sampled adults was conducted with a Nikon SMZ1500 stereomicroscope to distinguish main morphological characteristics. Then, specimens were identified according to the morphological description and identification key of *M. raddei* adults in the book named “Economic Insect Fauna of China (Volume 1) Cerambycidae” (Chen et al., 1959), and the original description of *M. raddei* (Blessig, 1872). Photographs of fresh specimens were taken with a UV‐C Optional Totally focused System (Beijing United Vision Technology Co. Ltd.) mounted on an Olympus CX31 microscope. All specimens were deposited in Insect Museum, Chinese Academy of Forestry, Beijing, China. The sampled populations were divided in two groups: (a) northern China, NC population group (Shandong Province Qixia City, SD; Inner Mongolia Ningcheng County, NMG; Liaoning Province Kuandian County, LN) and (b) southern China, SC population group (Anhui Province Hefei City, AH; Henan Province Xixia County, HN; Yunnan Province Pingbian Dawei Mountain, YNP; Yunnan Province Wenshan City, YNW) (Figure 1). ArcGIS platform version 10.2 was used to produce a distribution map based on the geographical coordinates of the localities. All samples were stored at −80°C before DNA extraction.

**TABLE 1:** Sampling information of seven geographical populations of *Massicus raddei* (Blessig) in China

Population Group	Province	Collection site	Population code	Longitude (°E)	Latitude (°N)	Sample size	Host species	Collection date
SC	Yunnan	Wenshan City	YNW	104.15	23.21	4	*Querues glauca*	May 2019
		Pingbian Dawei Mountain	YNP	103.41	22.56	2	*Quercus glauca*	August 2019
	Anhui	Hefei City	AH	117.10	31.50	12	*Quercus acutissima*	June 2019
	Henan	Xixia County	HN	111.28	33.17	4	*Quercus acutissima*	July 2019
NC	Shandong	Qixia City	SD	120.44	37.13	6	*Quercus acutissima*	August 2019
	Inner Mongolia	Ningcheng County	NMG	118.45	41.38	6	*Quercus mongolica*	August 2019
	Liaoning	Kuandian County	LN	125.11	40.44	6	*Quercus mongolica*	August 2019

### 2.2 DNA extraction, amplification, and sequencing

Genomic DNA was extracted from mesothorax muscles (approximately 0.1 g) after the epicuticle was removed, using the DNeasy Tissue & Blood Kit (QIAGEN) in accordance with the manufacturer's instructions. Extraction yield was measured using a Nanodrop‐1000 Spectrophotometer (DS‐11 Envivx). Subsequently, products were diluted to 30–40 ng/μL and stored at −20°C before polymerase chain reactions (PCR).

Based on the mitochondrial genome sequence of *M. raddei* (Wang et al., 2016), we designed specific primers for the partial sequences of the *COI*, *Cytb,* and *COII* using Primer version 3.0 (http://primer3.ut.ee/) (Kõressaar et al., 2018), and these primers were synthesized by Invitrogen Biotechnology Co., Ltd. Details about primer sequences and product size are given in Table S1.

Polymerase chain reactions (PCR) were performed using a S1000 Thermal Cycler (BIO‐RAD) in a total volume of 25 μl containing 12.5 μl 2× Green‐Mix, 1 μl forward and reverse primer (10 μmol/L), 1 μl genomic DNA, and 9.5 μl ddH_2_O. PCR amplification was employed with an initial denaturation at 94°C for 5 min, followed by 35 amplification cycles consisting of 95°C for 30, 30 s at a primer‐specific annealing temperature (52°C for *COI* gene, 53°C for *Cytb* and *COII* genes) and an extension at 72°C for 40 s, and then a final elongation at 72°C for 10 min. The products were visualized by 1% agarose gel electrophoresis and gel imaging system (UVP, Biolmaging Systems), and sequencing was implemented by Invitrogen Biotechnology Co., Ltd.

### 2.3 Mitochondrial DNA statistical analysis

Sequencing data were analyzed and edited using Geneious R11.1.5 (https://www.geneious.com; Kearse et al., 2012) and Bioedit version 7.2.5 (Clustal W method, default settings) (Hall, 1999). In order to corroborate that the correct gene fragments were obtained, sequence homology analyses were done using the BLASTN tool, which is available online from the NCBI (http://blast.ncbi.nlm.nih.gov.). Sequences of *COI* (679 bp), *COII* (369 bp), and *Cytb* (462 bp) of *M. raddei* were deposited in NCBI Genbank (Genbank accession numbers: *COI*, from MN796130 to MN796169; COII, from MN796170 to MN796209; *Cytb*, from MN796210 to MN796249). Subsequently, these three partial genes from the same individuals were concatenated to yield a combined sequence of 1,512 bp as a single locus because of the lack of recombination of mtDNA (Moraes et al., 2002). The nucleotide composition, number of conserved sites, variable sites, and parsimony informative sites of the three single partial gene sequences were then analyzed with Mega version 7.0 (https://www.megasoftware.net/citations).

### 2.4 Intrapopulation genetic diversity

Haplotypes were identified based on single and concatenated genes, respectively. Genetic diversity was estimated by number of haplotypes (n), haplotype diversity (*Hd*), nucleotide diversity (*Pi*), and the nucleotide average difference (*K*) for concatenated genes with the DnaSP version 5.0 (Librado & Rozas, 2009).

### 2.5 Genetic differentiation and genetic flow

Based on concatenated genes, pairwise fixation (*Fst*) and genetic flow indices (*Nm*) were estimated with DnaSP version 5.0 (Librado & Rozas, 2009). Pairwise average genetic distances between different populations were calculated by Kimura‐2‐parameter model with Mega version 7.0. Analysis of molecular variance (AMOVA) based on *Fst* (using haplotype frequencies) was conducted with 10,000 permutations to estimate the hierarchical genetic structure using the Arlequin version 3.5 (Excoffier & Lischer, 2010). Only two hierarchical levels were defined: (a) among populations and (b) within populations. Following the criterion for genetic differentiation of Wright (1978), we defined genetic differentiation as low for *Fst* < 0.05, moderate for 0.05 < *Fst* < 0.15, high for 0.15 < *Fst* < 0.25, and very high for *Fst* > 0.25 (Govindaraju, 1989). With reference to the criterion for gene flow values, we defined genetic flow as low for *Nm* < 1, high for 1 < *Nm* < 4, and very high for *Nm* > 4 (Boivin et al., 2004). The geographical distances were calculated according to longitude and latitude data using Geographic Distance Matrix Generator version 1.2.3 (Ersts, 2018). To examine whether any isolation‐by‐distance (IBD) effect occurred, matrices of pairwise genetic distances and geographical distances (km), matrices of genetic differentiation estimated by [*Fst*/(1 − *Fst*)], and the natural logarithm of geographical distance data (In Km) between all the sampling sites were analyzed for their degree of correlation by two mantel tests, with significance tests performed with 9,999 permutations. The analysis was implemented in GenAIEX 6.41 (Peakall & Smouse, 2012).

### 2.6 Phylogenetic relationships among populations and haplotypes

Applying Mega version 7.0 (https://www.megasoftware.net/citations.), ML phylogenetic analyses were used to identify major clades and evaluate the relationships among the seven populations by concatenated genetic sequences. An ML phylogenetic tree was constructed using General Time Reversible model with 1,000 bootstrap replications (Kumar et al., 2016). The Bayesian tree was obtained by running Mrbayes v3.1.2 (Ronquist & Huelsenbeck, 2003). The MCMC method was used to calculate 3 million generations, sampling once every 100 generations to ensure independence of sampling. The original 3,000 trees were discarded as burn in.

Haplotype networks of *M. raddei* were generated using a median‐joining algorithm in Network 5.0 (Bandelt et al., 1999), and the geographical distribution maps of haplotypes were visualized using PopART v1.7 (http://popart.otago.ac.nz/index.shtml. Leigh & Bryant, 2015) based upon private and concatenated genes, respectively. Applying Mega version 7.0 (https://www.megasoftware.net/citations), the optional alternative model (GTR + G) was used to construct Maximum‐Likelihood (ML) phylogenetic trees using haplotypes detected from private genes (*Cytb*, *COI*, *COII* genes) and concatenated genes. Bootstrap nodal support was evaluated from 1,000 bootstrap replicates. We aimed to examine whether there exists the high diversification between SC and NC population groups, and then further investigate the extent of nucleotide variation among different populations in each single gene sequences.

### 2.7 Demographic history and neutrality test

With the DnaSP version 5.0 software (Librado & Rozas, 2009), the Tajima's *D* and Fu's *Fs* values were estimated to test demographic expansion (Kimura, 1983; Tajima, 1989), and we assessed their significance with 1,000 permutations at levels of each geographical populations and overall population. The demographic history of whether *M. raddei* experienced a range expansion was also investigated by mismatch distributions of pairwise differences among sequences calculated with parametric bootstrapping (1,000 replications). The expected values were calculated assuming that sudden population growth model through coalescent simulations. According to simulations, demographically stable or admixed populations must present a multimodal distribution, whereas populations that have undergone a recent expansion generally exhibit a unimodal distribution (Harpending et al., 1998).

## 3 RESULTS

### 3.1 Sequence analyses

Based on the specimen identification, all sampling adults were determined as the adults of *M. raddei* (Figure S1). Forty partial *COI*, *Cytb,* and *COII* gene sequences of *M. raddei* with a length of 679, 462, and 369 bp were obtained, respectively. Nucleotide compositions among the seven geographical populations were basically consistent. The total content of A + T was much higher than that of G + C, which was in accordance with the feature of mitochondrial nucleotide composition (Jermiin & Crozier, 1994) (Table 2).

**TABLE 2:** Variation of *COI*, *Cytb,* and *COII* sequences of seven populations of *Massicus raddei* (Blessig)

Genes	Sequences length (bp)	C	V	Pi	S	A + T (%)	C + G (%)
*COI*	679	650	29	17	12	62.2	37.7
*Cytb*	462	430	32	8	24	65.4	34.6
*COII*	369	348	21	13	8	65.2	34.8

Note

C: conserved sites; V: variable sites; Pi: parsimony informative sites; S: singleton variable sites.

### 3.2 Genetic diversity

**FIGURE 2:**
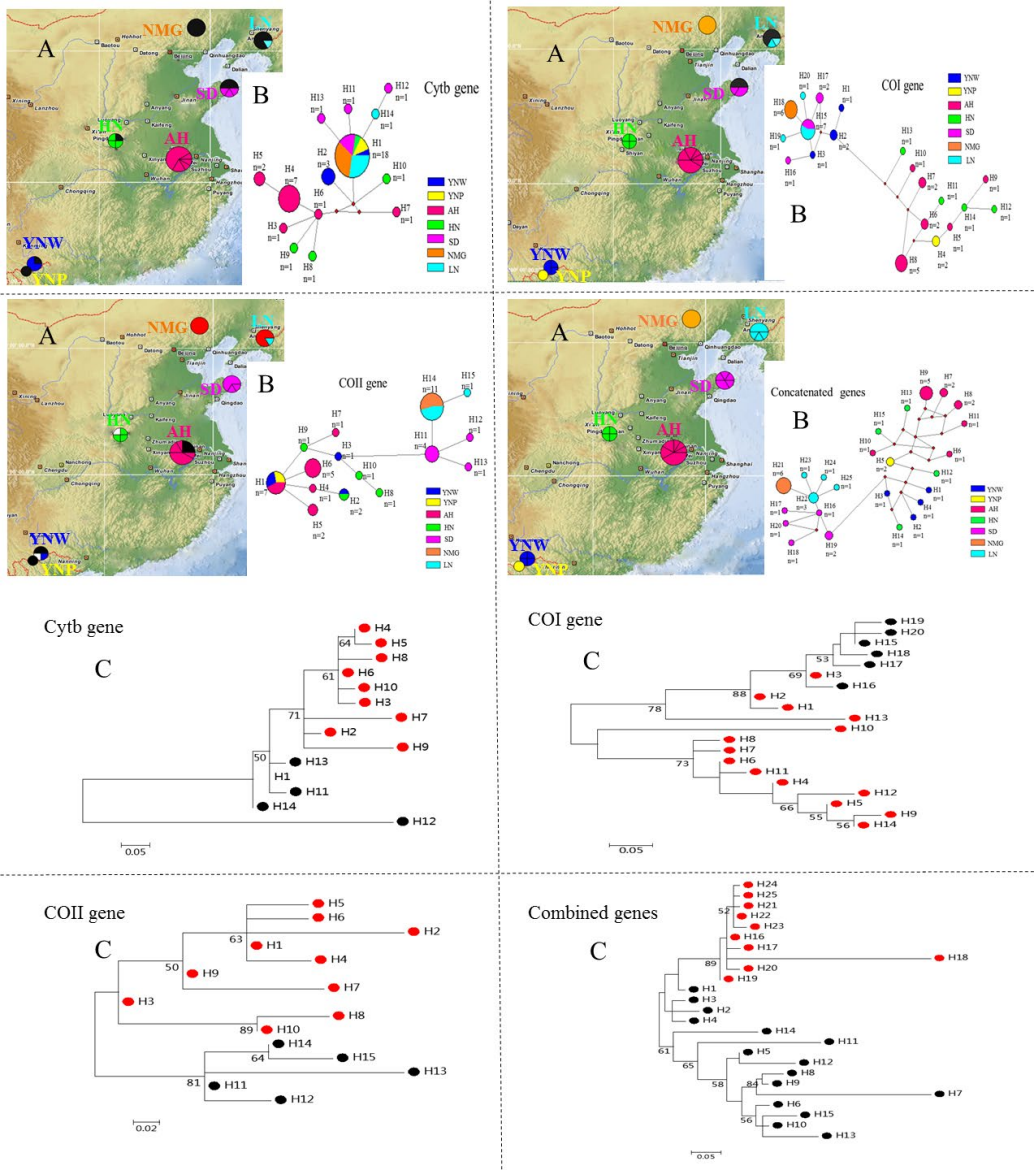
(A) Geographical distribution map of haplotypes detected from Cytb, COI, COII and concatenated genes. The population codes are labeled in different colors like the legend of the section B. The unique haplotypes only present in a population are marked in seven colors of the legend at right. One shared haplotype H1 and H15 obtained from Cytb and COI genes are both colored in black. Three shared haplotypes H1, H2, and H14 obtained from COII gene are colored in black, white, and red, respectively. (B) Median‐joining haplotype networks of 7 different populations of M. raddei based on Cytb, COI, COII and concatenated genes. Each circle represents a haplotype, haplotype code and number of specimen per haplotype are labeled near each circle. Each color corresponds to a geographical population. Seven populations are shown in the color legend at right, and for population code information, see Table 1. The red dots represent the mutational nodes. (C) Phylogenetic trees of haplotypes of M. raddei based on mtDNA COI, COII, Cytb and concatenated genes (Maximum‐likelihood method). Numbers below the branches are the bootstrap values estimated with 1,000 replications. Bootstrap values above 50% are shown. Haplotypes detected in NC (SD, NMG, and LN) and SC (HN, AH, YNW, and YNP) population groups are colored in black and red, respectively. The common haplotype H1 shared by six populations detected from Cytb gene is not colored

For the *Cytb* partial gene sequences, the haplotype network displayed a classic star‐like pattern with H1 as the center from which other haplotypes diverged outward, of which haplotype H1 might be an ancestral haplotype. The haplotype H1 was shared by six populations (LN, NMG, SD, HN, YNW, and YNP). Thirteen haplotypes, consisting of one shared haplotype and 12 exclusive haplotypes, were observed. Based on the *COI* partial gene sequences, only one common haplotype was shared between Shandong (SD) and Liaoning (LN) populations, while 19 unique haplotypes were detected. The *COII* gene sequences contained 15 haplotypes, comprised of three shared haplotypes and 12 unique haplotypes. Among these haplotypes, the two shared haplotypes in the SC population group were shared by the YNW, YNP, and AH (first haplotype) and YNW and HN (second haplotype). One common haplotype in the NC population group was shared with LN and NMG. Furthermore, 25 unique haplotypes without links to the shared or ancestral haplotypes were observed for concatenated gene sequences (Figure 2). From the concatenated genes, the average number of haplotypes (n) in each population was 3.571, ranging from 1 to 6, and the population as a whole showed a high haplotype and nucleotide diversity (*Hd* = 0.96; *K* = 15.85). In terms of a single population, haplotype diversity (*Hd*) ranged from 0.000 to 1.000. Nucleotide diversity (*Pi*) and the average number of nucleotide differences (*K*) ranged from 0% to 1.38% and from 0.00 to 20.83, respectively. Of these, the HN and YNW populations displayed the highest haplotype diversity, with each individual possessing one unique haplotype. HN population also developed the largest nucleotide diversity (0.0138) and differences (20.83) (Table 3).

**TABLE 3:** Genetic diversity parameters and neutrality tests among seven populations of *Massicus raddei* (Blessig) based on concatenated genes

Population	Number of haplotype	Haplotype diversity	Nucleotide diversity	Average number of nucleotide differences	Neutrality test and significance test	
Code	(*n*)	(*Hd*)	(*Pi*)	(*K*)	Tajima's *D*	Fu's *Fs*
YNW	4	1	4.00E−03	6	−0.84	−0.288
YNP	1	0	0	0	—	—
AH	6	0.82	5.00E−03	7.49	−1.22	2.181
HN	4	1	1.38E−02	20.83	−0.214	1.158
SD	5	0.93	6.10E−03	9.13	−1.248	0.667
NMG	1	0	0	0	—	—
LN	4	0.8	9.00E−04	1.33	−1.295	−1.252
Total	25	0.96	1.05E−02	15.85	−1.034	−2.500*

Note:

For population code information, see Table 1. In two neutrality tests, “*” represents statistical significance (*p* < .05). Insignificant values (0.05 < *p* < .10 or *p* > .10) are not labeled. “—” represents that no polymorphisms in sequences were provided to do neutrality tests.

### 3.3 Genetic differentiation and genetic structure

Pairwise *Fst* and *Nm* analyses were calculated for 21 pairs of populations with values ranging from 0.1104 to 1.0000, and 0.00 to 2.01, respectively. The results showed significant genetic differentiation and limited gene flow between almost all pairs of populations except for two pairs (AH and HN, and also SD and LN populations). Although AH and HN populations have developed moderate genetic differentiation, comparatively high genetic diversification was also observed between SD and LN populations. Based on the Nm values, frequent genetic flow was under a way between the two sets of populations, respectively (Table 4). Moreover, AMOVA results indicated that the larger proportion of molecular genetic variation was found among populations (62.97%), and the remaining variation came within populations (37.03%). Exact tests showed significant variation on two levels (*p* < .001) (Table 5).

**TABLE 4:** Genetic differentiation (*Fst*) and gene flow indices (*Nm*) among seven populations of *Massicus raddei* (Blessig) based on concatenated genes

Population code	YNW	YNP	AH	HN	SD	NMG	LN
YNW		0.07	0.15	0.6	0.34	0.09	0.12
YNP	**0.7857**		0.17	0.92	0.08	0	0.01
AH	**0.6246**	0.2138		**2.01**	0.11	0.04	0.06
HN	**0.2939**	**0.7638**	0.1104		0.36	0.2	0.24
SD	**0.4271**	**1**	**0.6863**	**0.4066**		0.54	**1.25**
NMG	**0.7391**	**0.96**	**0.8488**	**0.552**	**0.315**		0.17
LN	**0.6692**	**0.5954**	**0.8192**	**0.5137**	0.1664	**0.6**	

Note

For population code information, see Table 1. *Fst* values are below the diagonal and *Nm* values are above the diagonal. The *Fst* values in bold indicate great genetic differentiation between populations (*Fst* > 0.25) and the *Nm* values of bold type indicate frequent gene flow (*Nm* > 1).

**TABLE 5:** Analysis of molecular variance (AMOVA) of seven populations based on concatenated genes

Source of variance	*d. f*.	Sum of squares	Variance components	Percentage of variation (%)	*p* value
Among populations	6	201.392	5.544Va	62.97	.001
Within populations	33	107.583	3.260Vb	37.03	.001
Total	39	308.975	8.804		

Note

Va, Vb: Number of variance components.

**FIGURE 3:**
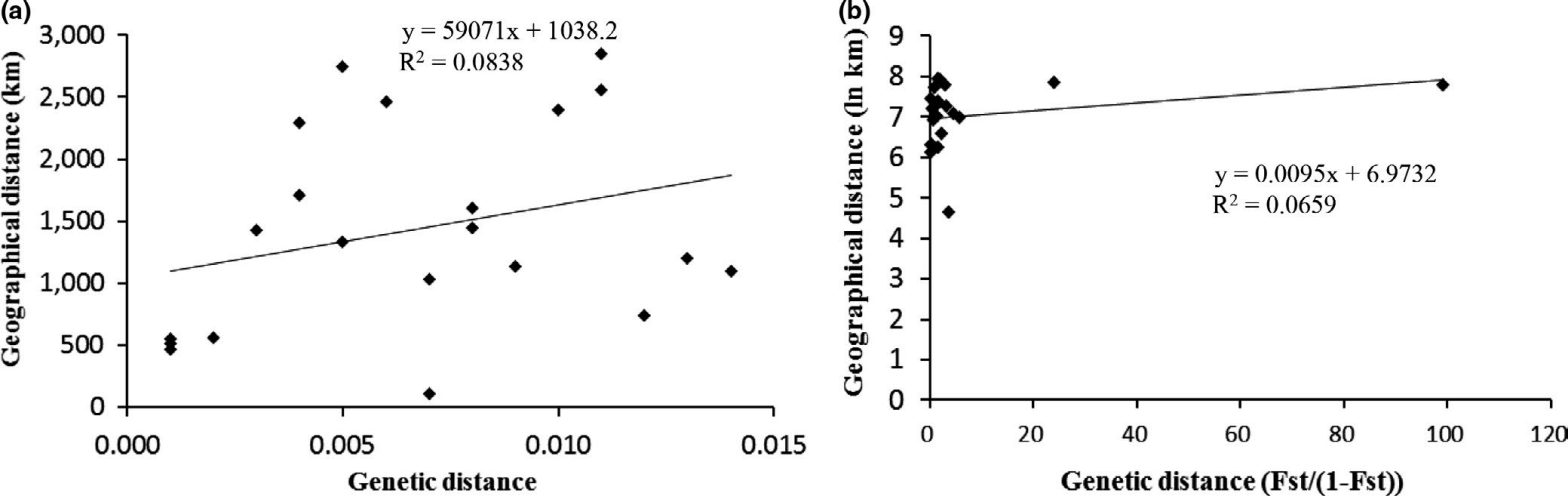
(A) The mantel test between genetic distance and geographical distance among seven spatially separated populations of *Massicus raddei*.(Blessig) (B) The mantel test between [Fst/(1‐Fst)] and the logarithm of geographical distance among seven spatially separated populations of *Massicus raddei* (Blessig)

Through two mantel tests, no significantly positive relationships between geographical distances and genetic diversification were found over seven geographical populations of China based on concatenated genes. *R* = .289, *p* = .138 and *R* = .257, *p* = .100 were detected between average pairwise genetic distances and geographic distances and between lnKm and *Fst*/(1 − *Fst*)) (Figure 3).

**FIGURE 4:**
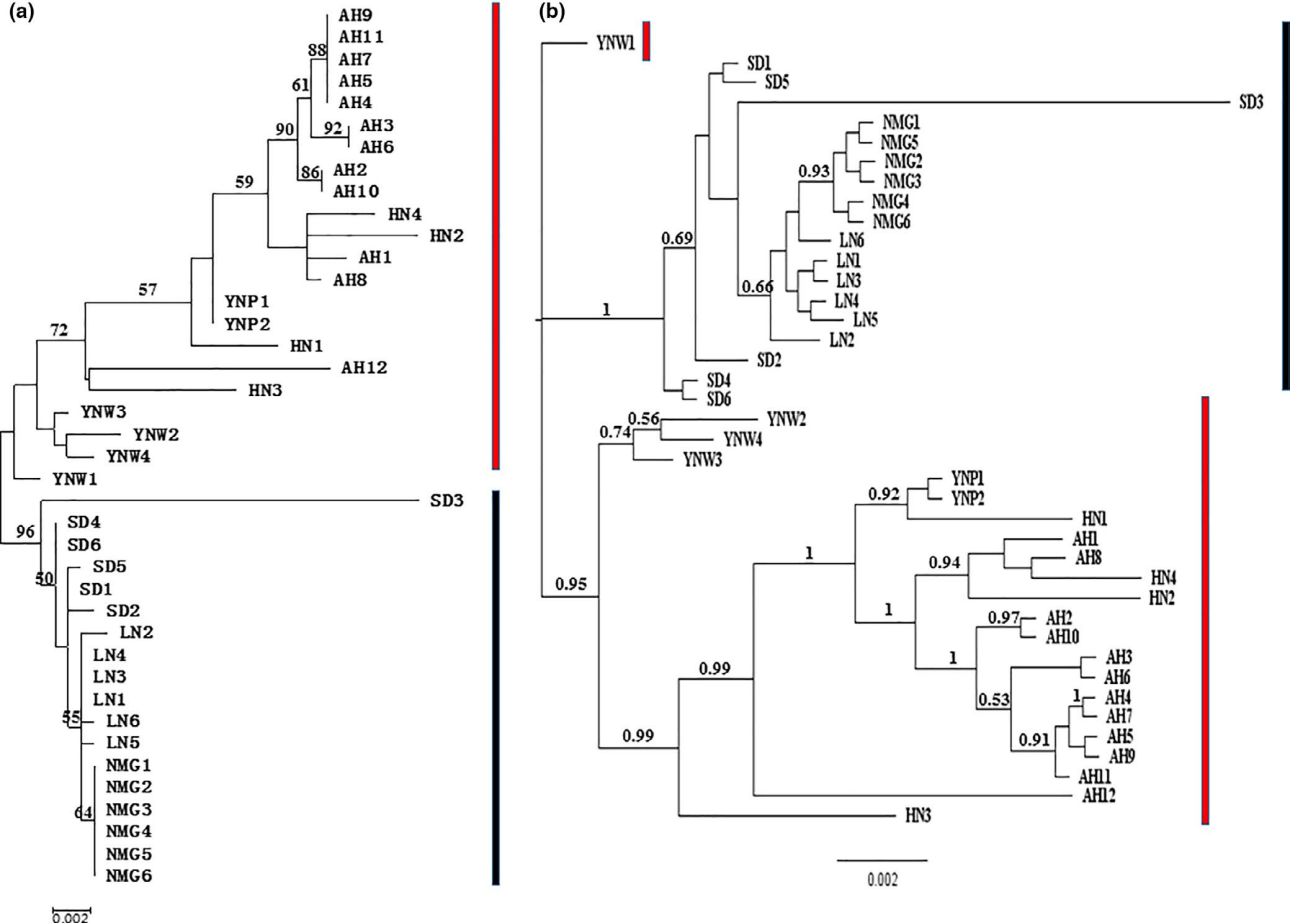
Unrooted ML (A) and BI (B) phylogenetic trees among seven populations based on concatenated genes. For population code information, see Table 1. Numbers above the nodes are bootstrap values and posterior probability values above 50%. SC and NC population groups are labeled by red and black lines, respectively

Based on single *COII* gene, the Median‐Joining haplotype networks and ML phylogenetic trees both displayed two apparent clades (SC and NC population groups) with strong bootstrap supports. Based on concatenated genes, SC and NC population groups were not divided into two apparent clusters. However, there was still an obvious division observed between SC and NC population groups despite of low bootstrap values in some branches. In contrast, the Median‐Joining networks and ML phylogenetic trees of the haplotypes based on single genes of *COI* and *Cytb* were not in the line with results obtained from *COII* and concatenated genes, which resulted from the lower nucleotide diversity and less genetic variation (Figure 2). Based on BI and ML phylogenetic trees, similarly, a relatively obvious genetic division was identified between SC and NC population groups in accordance with what haplotype networks exhibited. SC population group gathered into one cluster with comparatively high bootstrap values, in which NMG and LN populations were grouped into a clade first then combined with SD population. NC population group did not represent another distinct cluster with strong bootstrap values (Figures 2 and 4).

### 3.4 Demographic history

**FIGURE 5:**
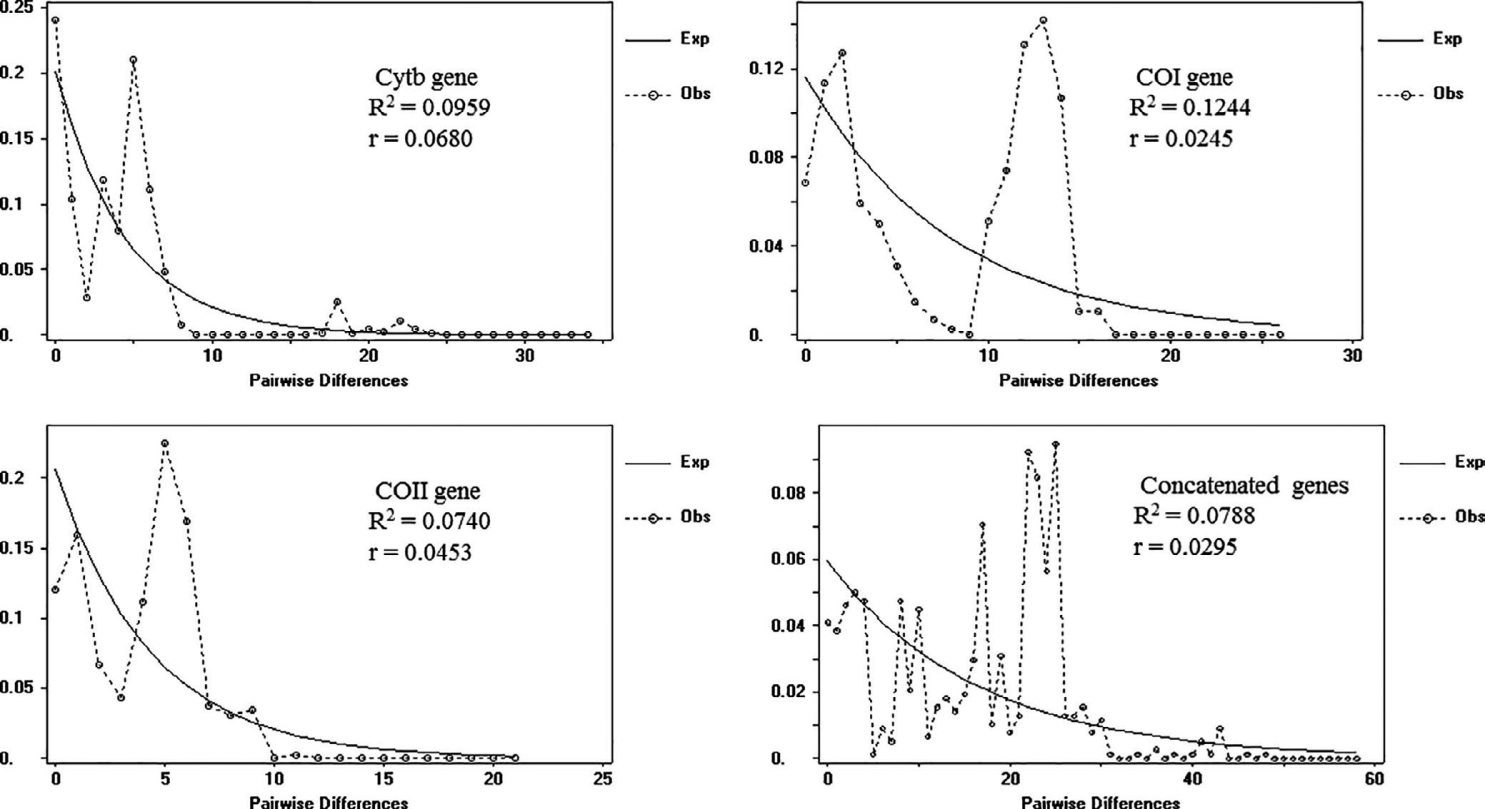
Observed and expected mismatch distribution analyses of entire samples of *Massicus raddei* (Blessig) in China based on mtDNA Cytb, COI, COII and concatenated gene sequences. The horizontal axis represents the number of pairwise differences, and the vertical axis shows relative frequency

All Tajima's *D* values of total and single populations based on concatenated genes showed negative and insignificant values (*p* > .10 or 0.05 < *p* < .10). Fu's *F* statistic of entire populations showed a significantly positive result with a value of −2.500 (*p* < .05), whereas at the single population level, insignificant values were observed (*p* > .10) (Table 3). Distributions of pairwise differences (mismatch distributions) of overall populations through concatenated and single gene sequences presented a multi‐peak distribution (Figure 5), suggesting that the seven geographical populations of *M. raddei* did not undergo any demographic expansion recently (Harpending et al., 1998; Rogers & Harpending, 1992). Insignificant values on the basis of Tajima’*D* and Fu's *Fs* also supported this interpretation.

## 4 DISCUSSION

The present study used three genes of mitochondrial DNA to provide basic molecular information to allow better insight into factors contributing to genetic diversification among populations and possible mechanisms triggering the evolution of different patterns of adult emergence of *M. raddei*. High genetic diversity and distinct genetic differentiation among sampled populations were identified through the mitochondrial analyses, and the assumption that particular periodic behaviors and outbreaks of *M. raddei* in Liaoning Province mainly result from the absence of genetic flow with other populations and local genetic isolation was preliminarily denied.

### 4.1 Genetic diversity

The largest nucleotide diversity and differences are detected in HN population, and four beetle specimens occupy one unique haplotype, respectively. All results provide a possibility that Xixia County might constitute the ancestral range of this species in Henan Province. The assumption requires more samples of Xixia County and adjacent areas to be further evaluated and verified. High number of unique haplotypes, lack of ancestral and shared haplotypes reveal that *M. raddei* are not invasive species being recently introduced, but how long they have settled in China poses a need for subsequent confirmation. Carter et al. (2009), Carter et al. (2010) identified the lower mtDNA haplotype diversity of *A. glabripennis* (Coleoptera: Cerambycidae) in invasive range comparing with native populations due to population bottleneck and genetic drift. 37 different mitochondrial haplotypes were obtained from a sample of 131 beetles in Asia, whereas only 12 unique haplotypes were detected in 258 beetles from North America. Javal et al. (2017) also observed the general pattern of decreasing exclusive haplotype frequency of invasive populations of *A. glabripennis* in comparison with Asian native populations. Three most common haplotypes are closely shared and dominate all invasive regions. Zheng et al. (2013) reported that large number of exclusive haplotypes might be indicators for native species, especially for insect species with low mobility. Kononov et al. (2016) identified the lower haplotype diversity of a fir bark beetle, *Polygraphus proximus* Blandford (Coleoptera: Scolytinae), following its invasion into Siberian and European parts of Russia from its native range. Men et al. (2017) also reported that *Dendrolimus kikuchii* Matsumura (Lepidoptera: Lasiocampidae) had similar genetic characteristics among various spatial populations, which might be native to southern China.

### 4.2 Genetic differentiation and structure

Among all populations of *M. raddei* investigated, most have developed strong genetic differentiation and a low level of genetic flow, and the major proportion of genetic diversification was found among populations. Although *M. raddei* adults have a moderate flight ability allowing short‐distance dispersal (Gao, 2001), together with anthropogenic larval migration by occasional transportation of infested timbers, while most sampling sites in the present experiment are far from each other and restrictions in the landscape permeability can reduce genetic exchange between habitat patches to a greater extent. As a result, there are few chances for movement among populations, and with the help from variation in regional microclimate, biology and genetic drift, significant genetic diversification has gradually developed. Usually, IBD effects are pronounced in moderately mobile species, but are weak in low‐ and high‐mobility species (Peterson & Denno, 1998). Among the populations of strong dispersers, extensive gene flow homogenizes populations across large geographic areas, whereas in sedentary species, limited gene flow allows nearly all populations to diverge even without the effects of geographical barriers. In intermediate dispersers, genetic homogeneity is achieved at small spatial scales, but limited dispersal ability reinforces genetic divergence over long distances (Peterson & Denno, 1998). We have reason to assume that there are positive correlations between genetic and geographic distances among populations of *M. raddei*. However, our current data were unable to obtain statistically significant results in mantel tests, which mainly resulted from our small sample size.

Genetic differentiation among populations is strongly correlated to various factors, such as a species’ dispersal ability, geographical barriers, ecological difference, and human‐mediated migration (Miller et al., 2003; Restoux et al., 2010; Sun et al., 2012; Tuda et al., 2014; Wang et al., 2015). As for genetic differentiation and genetic flow inches between pairwise populations, AH and HN populations were found to show the least genetic differentiation and frequent gene flow. Aside from comparatively shorter geographic distance between the two sites, abundant railway lines and highway routes have developed between Anhui and Henan provinces. Therefore, to a great extent, anthropic movement of infested wood or wood products provide routes for the hitchhiking of living adults and larvae, and this likely increased the genetic exchange between local populations. Additionally, the two local populations share the same host species *Q. accutisima*, possibly playing some roles in the development of lower genetic diversification. Although SD and LN populations are isolated by the Bohai Sea and infest different host species, comparatively frequent gene flow was also observed between these two populations, for which frequent marine timber transportation, similar oceanic monsoonal climates and adult dispersal by shipping may be responsible. Simultaneously, the LN population still had frequent gene flow with the SD population. So the absence of genetic exchange with other spatial populations and local isolation were not observed in the periodical population of Liaoning Province, which seems an unlikely explanation for its pattern of adult emergence. Alternatively, environmental factors might be plausible factors. Furthermore, high genetic differentiation and obvious genetic diversity among the seven populations might conversely determine great variance in fecundity, larval capacity of degradation and nutrient acquisition along with adaptability to extreme environmental conditions, which further leads to the differences of regional population density and destructive levels to oak forest ecosystems.

With reference to phylogenetic analyses of haplotypes and concatenated sequences, distinct genetic differentiation is detected between NC and SC population groups. Indeed, different geographic populations of *M. raddei* attack various oak tree species (EPPO, 2018). *Q. glauca* and *Q. acutissima* are commonly infested in southern populations of China, whereas *M. raddei* regularly exploits *Q. mongolica* and *Q. liaotungensis* in northern regions of China. Within SC and NC population groups, pairwise populations AH and HN, NMG and LN shared the same clade with lower genetic distance. Coincidently, two pairs of populations infest the same host species *Q. acutissimam* and *Q. mongolica*, respectively. So, a host‐associated differentiation (HAD) might occur among spatial populations of *M. raddei*. Previous studies have well documented that among polyphagous insects, ecological divergence occurs as a consequence of the selection pressure imposed by the host traits. Even in the absence of physical barriers, different host resources can act as the selection basis to form new ecotopes of herbivores and subsequently strengthen their genetic differentiation. Consequently, geographical populations exploiting different resources might be genetically distinct (Abrahamson et al., 2001; Ferrari et al., 2012; Forbes et al., 2017; Medina et al., 2012; Ruiz‐Montoya et al., 2003). However, few previous studies have reported that HAD phenomenon has been observed among wood‐feeding longhorn beetles, but for *A. glabripennis*, another wood‐boring pest, some experiments demonstrated that host trees can affect the insect's gut microbial community composition, the level of cellulase activity, and the expression level of digestive and detoxification genes (Erin et al., 2018; Geib et al., 2009). Therefore, it is presumed that similar effects are likely to occur among *M. raddei* larvae which live in various oak tree species, and that host plant‐associated genomic differentiation will evolve over time. Unexpectedly, some exceptions were also detected. Although SD population infest *Q. acutissimam* the same as AH and HN populations, distinct genetic diversification was obtained with other two spatial populations. Frequent gene flow with LN population, restricted genetic exchange with southern populations by geographical barriers and limited human‐aided pathways, might eventually shape the phylogeography. YNW and YNP populations infest common host species *Q. glauca* and are only isolated by comparatively shorter geographical distance (100 km), obvious HAD phenomenon was still not present between them. Recent invasive events or local microclimate might be as the cause, which needs more samples of Yunnan Province and adjacent areas to facilitate a more systematic investigation.

*Massicus raddei* have a wide natural distributional range, but only have developed high population density and reached destructive damage to forest ecosystems in Liaoning and Jilin provinces. By coincidence, among those regional populations, a periodic (once per 3 years) adult emergence phenology was also observed (EPPO, 2018). It is therefore assumed that there exists a causal relationship between the particular pattern of adult emergence and serious infestation. Previous studies have proposed that, within an insect species, the populations with annual emergence are considerably less dense than periodical populations (Bulmer, 1977; Lloyd & Dybas, 1966; Martin & Simon, 1990), and extraordinarily high densities and epidemic outbreaks in *M. raddei* populations of northeastern China are likely to be attributed to predation avoidance or predator satiation achieved by periodic behavior (Williams & Simon, 1995). In the present study, the assumption that serious periodic outbreak of population in Liaoning Province might be a result of local evolution in the absence of gene flow was preliminarily ruled out. Future researches should pay more attention on its biology, host‐parasitoid interaction, and climatic factors, which may trigger proto‐periodicity and favor the development and perfection of periodicity, in order to clarify the origin and maintenance mechanisms behind this interesting ecological phenomenon.

In conclusion, the present work, a preliminary attempt to investigate population structure across distributional areas of *M. raddei* in China. These results not only expand our genetic knowledge of this borer species, but also provide some new perspectives and theoretical basis for subsequent specialized studies in some regional populations. For instance, all spatial populations of Henan Province can be regarded as one research focus to evaluate the possibility of Xixia County as the ancestral center. More local populations of Shandong and Liaoning provinces should be collected to verify frequent gene flow and the lack of local genetic isolation between periodical and non‐periodical populations once again, and then provide more detailed information to obtain a comprehensive understanding of contributing ecological and artificial factors shaping the phenomenon. Moreover, future research employing more extensive samples and covering wider distributional sites, along with additional mitochondrial and nuclear molecular markers are needed to allow a better understanding of genetic structure among populations all over China.

## CONFLICT OF INTEREST

None declared.

## AUTHOR CONTRIBUTIONS

**Xiaoyi Wang:** Methodology (lead); writing – review and editing (equal). **Yufan Zhang:** Data curation (equal); investigation (lead); writing – original draft (lead). **Atif Manzoor:** Software (supporting); writing – review and editing (supporting).

## Supporting information

Figure S1

Table S1

## Data Availability

DNA sequences were deposited in Genbank. *COI* gene accession numbers: MN796130–MN796169; *COII* gene accession numbers: MN796170–MN796209; *Cytb* gene accession numbers: MN796210–MN796249. The specially designed primers aimed at *COI*, *COI,I* and *Cytb* genes of *M. raddei*, and a photograph of adult of *M. raddei* were uploaded as Table S1.
