# Supplementary figures and images for: Mitochondrial DNA analysis reveals spatial genetic structure and high genetic diversity of Massicus raddei (Blessig) (Coleoptera: Cerambycidae) in China
Source: Ecol Evol. 2020 Oct 1;10(20):11657–70. doi: 10.1002/ece3.6799 (PMC7593171; doi:10.1002/ece3.6799)

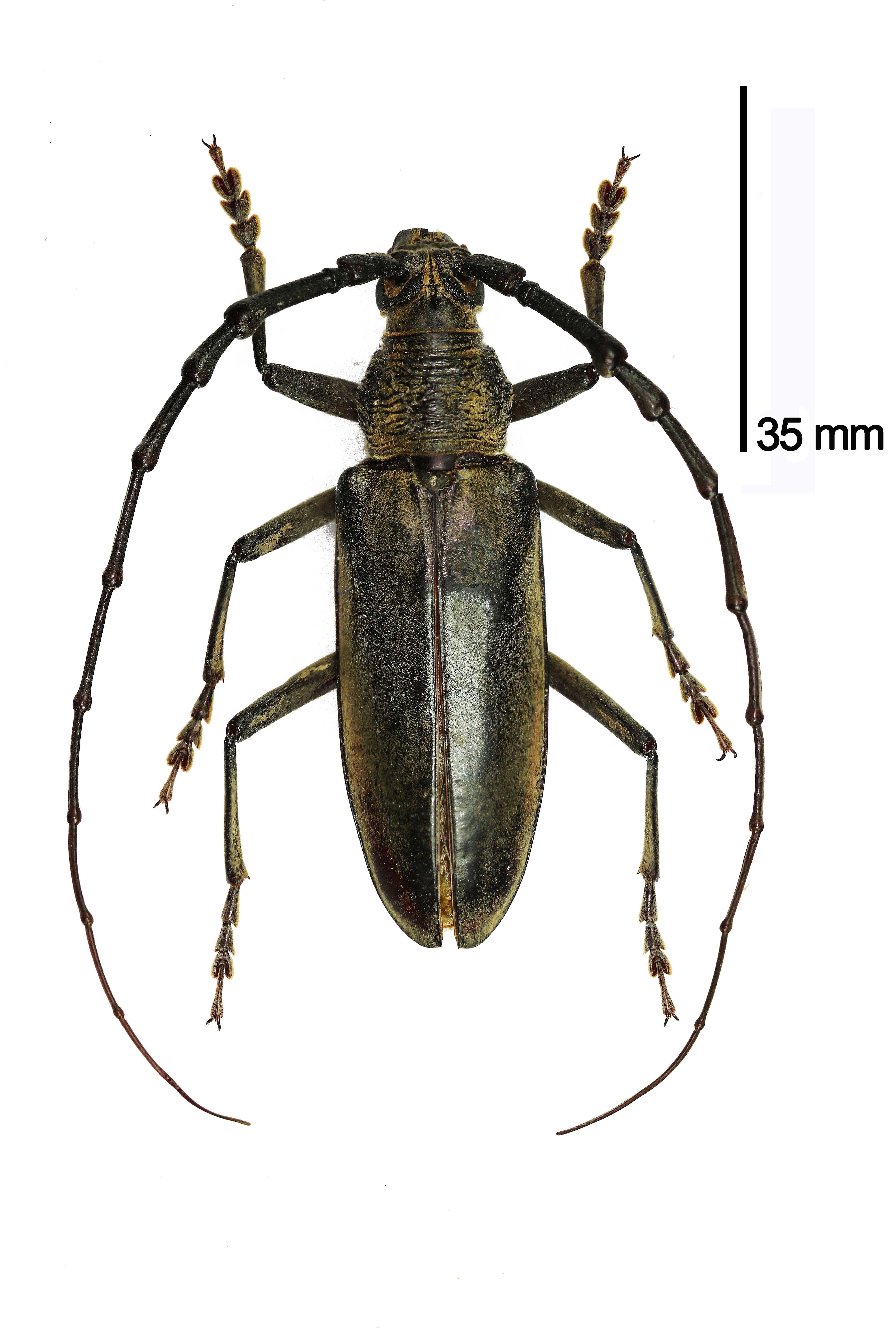

Supplement: Supplementary file 1 — Figure S1 [file ECE3-10-11657-s001.tif]
