# Supplementary material for: Mitochondrial DNA analysis reveals spatial genetic structure and high genetic diversity of Massicus raddei (Blessig) (Coleoptera: Cerambycidae) in China
Source: Ecol Evol. 2020 Oct 1;10(20):11657–70. doi: 10.1002/ece3.6799 (PMC7593171; doi:10.1002/ece3.6799)
Supplement: Supplementary file 2 — Table S1 [file ECE3-10-11657-s002.docx]

| Genes | |  | Primer sequences (5’-3’) | Product size (bp) |
| --- | --- | --- | --- | --- |
| COI |  | F: AATCGGAGGATTCGGAAACT  R: TCCTGTTGGCACTGCAATTA | 730 |  |
| CYTB |  | F: CGGGCGAGGAATTTATTACA  R: CTGGAGTAACGAGGGGATTG | 503 |  |
| COII |  | F CATACCTGCTGTGACTCTAGTATTT  R: CTGTGATTTGCTCCACAGATTTC | 414 |  |

# SUPPLEMENT

**Table 1** Special PCR primers aimed at COI, CYTB and COII genes of *Massicus raddei* (Blessig)
